# Glioma patients in outpatient care—optimization of psychosocial care in neuro-oncological patients (GLIOPT): Protocol for a cluster randomized controlled trial

**DOI:** 10.1186/s13063-020-04321-2

**Published:** 2020-05-27

**Authors:** Mirjam Renovanz, Melina Hippler, Martin Voss, Jens Wehinger, Almuth F. Keßler, Jens Gempt, Minou Nadji-Ohl, Carolin Weiß Lucas, Marion Rapp, Martin Misch, Jan Coburger, Marcus Mehlitz, Jürgen Meixensberger, Naureen Keric, Ghazaleh Tabatabai, Maria Blettner, Melanie Schranz, Susanne Singer

**Affiliations:** 1 Department of Neurology & Interdisciplinary Neuro-Oncology, University Hospital Tübingen, Tübingen, Germany; 2 Hertie Institute for Clinical Brain Research, Eberhard Karls University Tübingen, Tübingen, Germany; 3 Center for Neuro-Oncology, Comprehensive Cancer Center Tübingen-Stuttgart, University Hospital Tübingen, Eberhard Karls University Tübingen, Tübingen, Germany; 4 Department of Neurosurgery, University Hospital Tübingen, Tübingen, Germany; 5 Dr. Senckenberg Institute of Neurooncology, University Hospital Frankfurt, Goethe University, Frankfurt, Germany; 6 Department of Neurology, Clinic Ludwigsburg, Ludwigsburg, Germany; 7 Department of Neurosurgery, University Hospital Würzburg, Würzburg, Germany; 8 Department of Neurosurgery, Klinikum Rechts Der Isar, Technical University Munich, Munich, Germany; 9 Department of Neurosurgery, Klinikum Stuttgart, Katharinenhospital (KH), Stuttgart, Germany; 10 Center for Neurosurgery, University Hospital Cologne, Faculty of Medicine, University of Cologne, Köln, Germany; 11 Department of Neurosurgery, University Hospital Düsseldorf, Düsseldorf, Germany; 12 Department of Neurosurgery, Charité - University Medical Center Berlin, Berlin, Germany; 13 Department of Neurosurgery, University Hospital Ulm, Ulm, Germany; 14 Department of Neurosurgery, Klinikum Barmherzige Brueder Trier, Trier, Germany; 15 Department of Neurosurgery, University Hospital Leipzig, Leipzig, Germany; 16 Department of Neurosurgery, University Medical Center Mainz, Mainz, Germany; 17 Institute of Medical Biostatistics, Epidemiology, and Informatics (IMBEI), University Medical Center Mainz, Mainz, Germany

**Keywords:** Distress, Psychosocial care, Supportive care needs, Assessment, Primary brain tumor patients, High-grade glioma

## Abstract

**Background:**

Patients with high-grade gliomas (HGG) often suffer from high distress and require psychosocial support. However, due to neurological and neurocognitive deficits, adequate assessment of distress and support needs remains challenging in clinical practice. The objective of the present study is to investigate whether a systematic implementation of signaling questions into the routine outpatient consultation will be helpful to bridge this gap.

**Methods/design:**

This is a multicenter cluster randomized study with two arms. Randomization is done on a cluster level with 13 hospitals providing regular neuro-oncological outpatient services conducted by neurologists and/or neurosurgeons. The intervention will include an assessment of psychosocial distress of patients in doctor–patient conversation compared to assessment of psychosocial distress via questionnaire (control, standard of care).

In total, 616 HGG patients will be enrolled. The outcome will be the number of HGG patients with increased psychosocial distress who receive professional support from psychosocial services.

Secondary endpoints are inter alia number of patients reporting psychosocial distress and unmet needs detected correctly by the respective method; quality of life; psychological well-being and burden of the patients before and after doctor–patient consultation; as well as the length of the doctor–patient consultation.

**Discussion:**

Patients with HGG are confronted with an oncological diagnosis and at the same time with high symptom burden. This often leads to distress, which is not always adequately recognized and treated. So far, only a limited number of adequate instruments are available to assess HGG patient’s distress. Yet, an adequate care and support network might facilitate the course of the disease and tumor therapies for patients. Our hypothesis is that an assessment conducted directly by attending doctors and in which the doctors talk to patients with HGG will be more effective than an assessment via a questionnaire, leading to better identifying patients in need of support. This may lead to an improvement of health care in these patients. Further, this method might be implemented also in other brain tumor patients (e.g., patients with brain metastases).

**Trial registration:**

German Clinical Trials Register, DRKS00018079. Registered on 3rd September 2019.

## Background

Neuro-oncological diseases, especially the diagnosis of a high-grade glioma (HGG), are associated with psychosocial burden. Patients face not only an oncological disease but also neurological symptoms resulting in changes of cognitive, role, and social functioning. Patients therefore often require support in terms of socio-legal counseling, rehabilitation, psychological support, and support regarding palliative care aspects [1]. Topics such as providing adequate psychosocial and supportive care in order to maintain quality of life are part of the available guidelines for the provision of neuro-oncological care [1]. As glioma patients suffer from cognitive impairment early during the disease trajectory [2, 3], adequate assessment of unmet needs and a regular psycho-oncological screening are recommended in the requirements for certification of Comprehensive Cancer Centers. However, an essential aspect is not only to record the supportive care needs but also to provide care.

In order to identify cancer patients in need of support, several psychosocial screening instruments such as the Distress Thermometer (DT) have been developed [4, 5]. The DT consists of a numerical analogue scale assessing distress and a problem list for assessing support needs. It has been validated for brain tumor patients and seems to be a reliable tool to identify burdened patients [4]. However, screening instruments have been rarely adapted to the diverse needs of neuro-oncological patients so far [6–8].

Especially as HGG patients suffer from neurocognitive deficits during their disease trajectory caused by the disease itself and the treatment, they may not always be able to complete the questionnaires [6, 9, 10]. Therefore, either adaption of instruments to the needs of neuro-oncological patients or alternative assessment approaches are required. As an alternative, we developed questions that can be asked during the doctor–patient consultation and that are understandable also for patients in reduced clinical condition, with neurocognitive deficits and/or physical restrictions.

Whether integrating these screening questions into the doctor–patient consultation (intervention arm) results in better identification of patients in need of psychosocial support compared to care as usual (CAU; comparison arm) is the primary research question of this trial.

### Objectives and hypothesis

This study was designed to assess whether the psychosocial assessment in patients with HGG can be improved by face-to-face assessment during doctor–patient consultation.

The following aims are addressed:

- First, we compare the percentage of HGG patients in distress receiving adequate psychosocial care
- Second, we compare the percentage of HGG patients correctly identified as distressed in the two trial arms

We hypothesize that more patients will be adequately assessed regarding distress during the doctor–patient consultation (intervention arm) than by the questionnaire assessment (CAU arm). We further hypothesize that HGG patients assessed in the intervention arm will more frequently receive support than the others. The PICO scheme for this trial is as follows:

- Patients: Patients with high-grade glioma
- Intervention: Assessment of distress in doctor–patient consultation
- Comparison: Assessment of distress via questionnaire
- Outcome: Proportion of patients with high levels of distress who receive psychosocial care by specialized services

### Primary and secondary endpoints

The primary endpoint is the proportion of patients with increased psychosocial stress who receive professional support from psychosocial services (psycho-oncology, social services, cancer counseling centers, palliative care with psychosocial support).

Secondary endpoints are number of patients reporting psychosocial stress; number of patients receiving psychosocial care through specialized services such as psycho-oncology, social services, cancer counseling centers, palliative care, etc.; number of patients referred to care structures; quality of life; need for support; psychological well-being and burden of the patients before and after doctor–patient consultation; and duration of the doctor–patient consultation.

### Preliminary work

This is the first multicenter study with the aim to improve the health care situation of patients with HGG. We conducted several observational studies before that led to the research question of the GLIOPT trial. Hereby we found that:

1. Some questionnaires do not fit to the requirements of HGG patients [6, 11].
2. Patients in reduced clinical condition with high unmet needs may be missed by a questionnaire or tablet assessment [8, 12].
3. We can use “signaling items” probably indicating patients in need of support [13]. Applying these items in a face-to-face conversation between doctor and patient was possible. It seems, therefore, to be an alternative approach for distress assessment in this patient group.

Based on these studies, we developed the research question whether psychosocial care could be improved by face-to-face needs assessment during doctor–patient consultation compared to screening for distress using a questionnaire.

#### Development of “signaling items” for use in doctor–patient conversation

We developed questions regarding the burden and supportive care needs of glioma patients by a literature search, an expert survey, and patient interviews [14]:

- Literature search: The research team analyzed the literature with regard to possible questions identifying supportive care needs in a doctor–patient consultation.
- Expert survey: Doctors who regularly see neuro-oncological patients were interviewed with regard to the importance of the questions identified from the literature.
- Pretesting: The preliminary questions were pilot-tested with ten patients. Afterwards, the questions were adapted accordingly.
- Patient interviews: Fifty patients were assessed using a structured interview. They estimated the importance of items and were asked to add missing items and to select the most important items of the preliminary questions. On the basis of the expert survey and patient interviews, three items/fields were identified by a weighted scoring procedure: psyche, body, and cognition.

#### Feasibility analyses of the three questions in clinical routine

The developed questions were applied in comparison to the questionnaires (EORTC QLQ-C30 + BN20, DT) in patients at different stages of the disease—perioperatively as well as in the outpatient setting [15]. Interviews were documented and the questions according to the results further adapted. The three questions are:

1. Has your mood worsened due to the disease?
2. Do physical changes due to the disease, such as numbness, weakness or feeling exhausted more quickly, put a strain on you?
3. Has your mental capacity worsened as a result of the disease, making it harder for you to concentrate or remember things, for example?

## Methods/design

### Study design

**Fig. 1:**
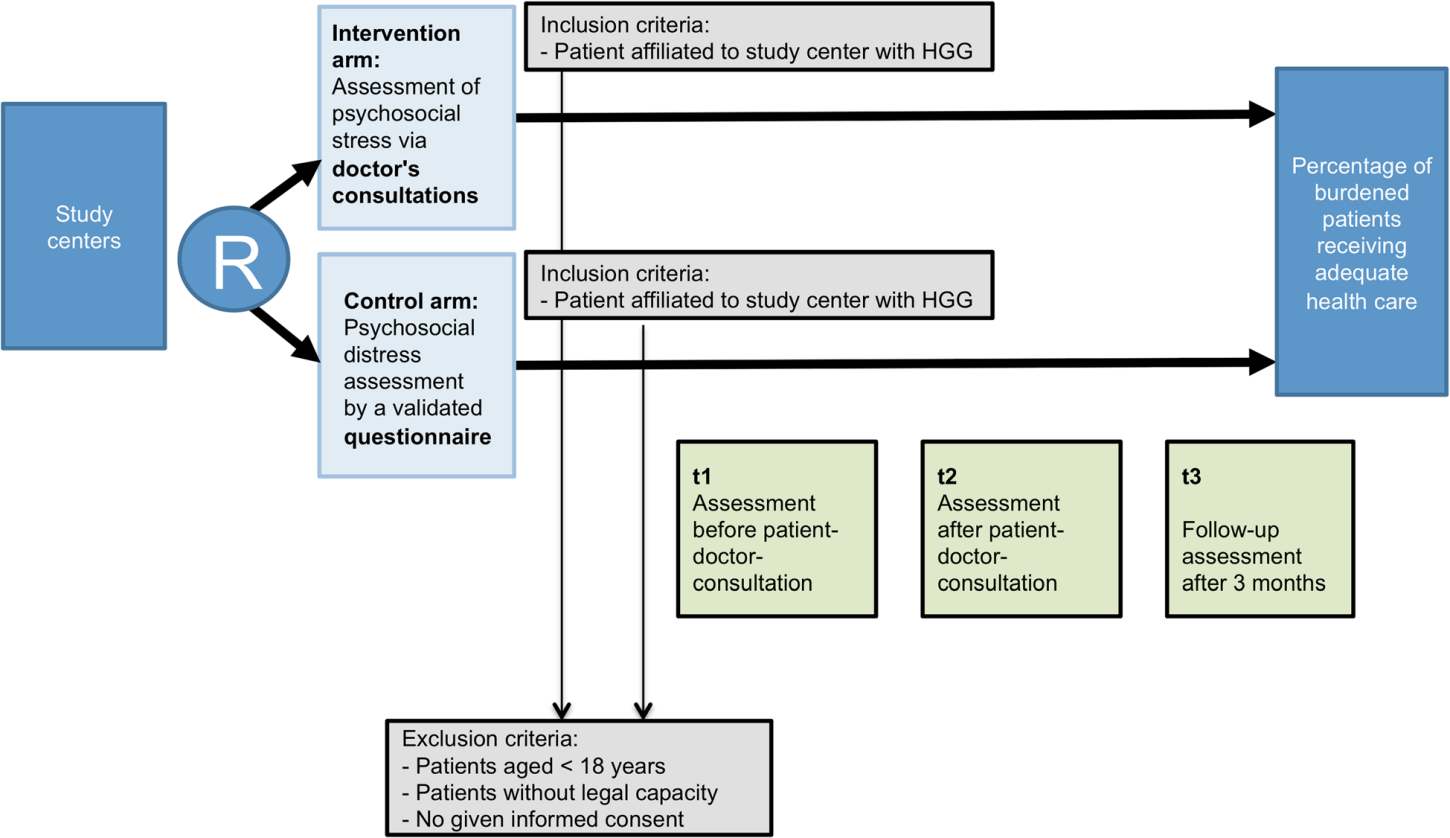
Course of the study

The study is a cluster-randomized, controlled, non-blinded, multicenter study with two parallel groups to be compared (Fig. 1). The study investigates the effects of two different ways of assessing psychosocial distress in outpatient HGG patients.

The clusters are the institutions (hospitals) participating in this trial (*n* = 13). In intervention arm hospitals, distress is assessed within the doctor–patient consultations. In CAU hospitals distress is assessed with the DT.

#### Setting

**Fig. 2:**
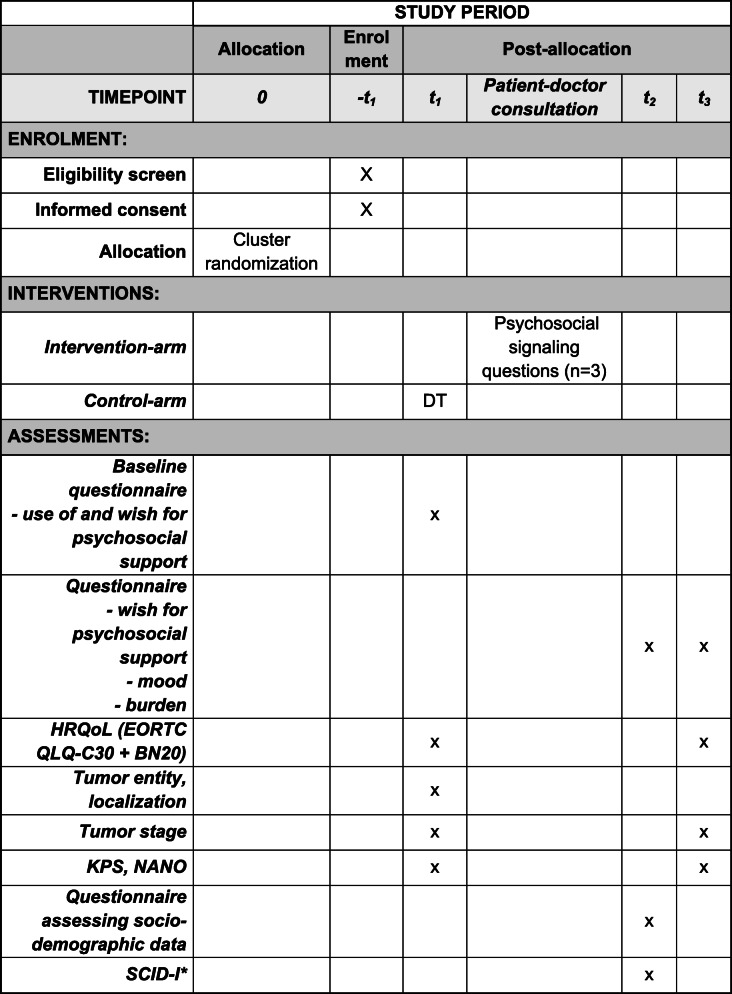
GLIOPT SPIRIT figure. *SCID* Structured Clinical Interview for DSM (Diagnostic and Statistical Manual of Mental Disorders), *KPS* Karnofsky Performance Status, *NANO* Neurologic Assessment in Neuro-Oncology, *EORTC QLQ-C30 + BN20* European Organization of Research and Treatment of Cancer Core Questionnaire (EORTC QLQ-C30) with its brain module (BN20), *DT* Distress Thermometer

Patients with HGG will be recruited from 13 geographically dispersed neuro-oncological centers throughout Germany (Tübingen, Mainz, Frankfurt, Ludwigsburg, Würzburg, München, Stuttgart, Köln, Düsseldorf, Berlin, Ulm, Trier, Leipzig). The time course is displayed in Fig. 2.

#### Randomization

The allocation to the two study arms is randomized. Through cluster randomization, the study is easier to implement into clinical routine compared to individual patient randomization. An external statistician who is not involved in treatment, planning, or evaluation of the study performed the randomization (Center for Clinical Studies in Mainz, Germany).

### Participants and procedures

#### Eligibility criteria

- Clusters: Departments as clusters were included, when 1) a neuro-oncological outpatient service was present with 2) regular weekly consultation hours and 3) conducted by neurologists or neurosurgeons.
- Patients: We include patients with a HGG (glioblastoma WHO°IV, anaplastic astrocytoma WHO°III, anaplastic oligodendroglioma WHO°III) aged 18 years and older with the ability to give informed consent. Patient exclusion criteria are absence of written informed consent and inability to understand the German language.

#### Screening and informed consent procedure

**Fig. 3:**
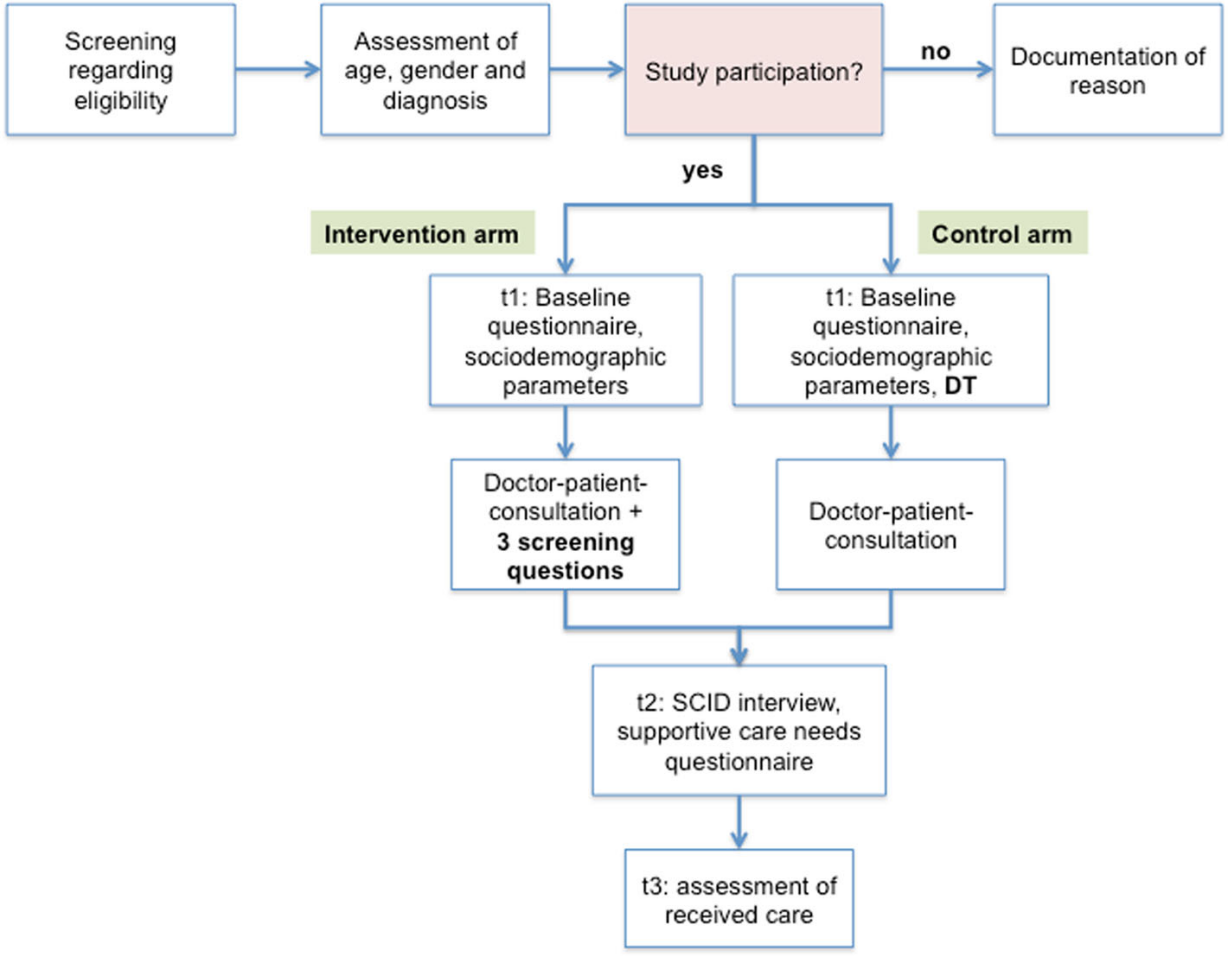
Recruitment procedure and flow of participants through the study. *DT* Distress Thermometer, *SCID* Structured Clinical Interview for DSM (Diagnostic and Statistical Manual of Mental Disorders)

The study coordinator or research assistant of every study center will screen the patients scheduled in the outpatient department to assess their eligibility. Then the study nurse will give each eligible patient a brief overview of the study and ask whether or not he or she would be interested in participation. The consent to participate in the study goes along with signing the consent form. In addition to the patient information, each patient receives a copy of the consent form. From decliners of the study, gender, tumor diagnosis, age, and the reason for decline will be documented (Fig. 3).

#### Clinical information assessed in the study

The following information is documented in both arms: location of the tumor, clinical condition defined by Eastern Co-operative of Oncology Group (ECOG) status and Karnofsky Performance Score (KPS), stage of disease (initial diagnosis/recurrence), neurological deficit defined by Neurologic Assessment in Neuro-Oncology (NANO) scale [16], time since initial diagnosis, age, and gender. Furthermore, patients will provide demographic information such as professional and financial situation, family status, and current care situation.

#### Conditions of the intervention arm

Psychosocial distress is measured by three questions that emerged as important in a preliminary study. The three questions are:

1. Has your mood worsened due to the disease?
2. Do physical changes due to the disease, such as numbness, weakness, or feeling exhausted more quickly, put a strain on you?
3. Has your mental capacity worsened as a result of the disease, making it harder for you to concentrate or remember things, for example?

On the basis of the patient’s answers, the doctor offers psychosocial care, if indicated and desired by the patient, and refers the patient to the appropriate specialist services, e.g., psycho-oncological service, clinic social service, outpatient cancer counseling centers, physiotherapy, established psychotherapists, etc.

#### Conditions of the control arm

In the control arm, psychosocial distress is assessed using the DT, which can be evaluated within a few minutes, so that the results in the control group can also be discussed during the doctor–patient consultation. If there is an obvious need for treatment or if a patient requires psychosocial support, the referral is carried out analogously to the intervention condition.

#### Psychosocial care in both arms

Depending on the urgency, care must be provided quickly in both arms, i.e., within a few days to a maximum of 2 weeks, depending on the capacity of the respective cooperating services (psycho-oncological, social-legal, etc.).

### Instruments applied in the study

#### Before the doctor–patient consultation, t1

First patients complete a baseline questionnaire (t1) assessing use of and wish for psychosocial support using an adapted version of a self-developed questionnaire [17]. Further, the European Organization of Research and Treatment of Cancer Core Questionnaire (EORTC QLQ-C30) with its brain module (BN20) will be applied. It is a self-assessment tool to evaluate cancer patients’ quality of life. Validity and reliability have been previously tested in several clinical studies [18].

Patients in the control group complete additionally the DT. This is a self-reporting screening instrument, measuring psychosocial distress by a numerical rating scale accompanied by a 40-item list with problems from different areas of life, e.g., practical, family, emotional problems, etc. [5, 19].

Afterwards, the patients have the doctor–patient consultation. In the intervention group, the three questions, described above, are asked during doctor–patient consultation instead of using the DT (Table 1).

**Table 1:** Operationalization of the outcomes and measurement points shown by outcomes

Outcome	Measure	t1	t2	t3
*Primary outcome: Psychosocial care through specialized services*				
Inpatient psycho-oncological service	Operation and procedure codes in the clinic information system or self-developed questionnaire			x
Cancer Counseling Center	Self-developed questionnaire			x
Inpatient social service	Operation and procedure codes in the clinic information system or self-developed questionnaire			x
Outpatient psychotherapy	Self-developed questionnaire			x
Neuropsychology	Medical records or operation and procedure codes in the clinic information system			x
Rehabilitation	Self-developed questionnaire or data derived from medical records			x
*Secondary outcomes*				
Percentage of patients that can be assessed for psychosocial distress	Participation rate in intervention arm vs control arm		x	
Length of doctor–patient consultation	In minutes (documentation by clinicians in the study worksheet)		x	
Emotional functioning/distress	EORTC QLQ-C30, emotional functioning scale	x		x
Health-related quality of life	EORTC QLQ-C30, global scale and BN20, all scales	x		x
Support requested	Self-developed questionnaire	x	x	x
Appointments arranged in specialized services	Documentation by clinicians in the study worksheet		x	
Direct costs of support	Calculated on the basis of the patient–doctor consultation duration			x
Accuracy of the screening method	Comparison of the screening results regarding unmet needs (intervention vs control arm) with a detailed diagnosis based on SCID interview		x	
*Evaluation of protocol adherence*	Questions to patients: whether they have completed a DT (control arm) or whether the doctor has inquired about their psychosocial condition (intervention arm)		x	

*Abbreviations*: *EORTC QLQ-C30* European Organization of Research and Treatment of Cancer Quality of Life Core Questionnaire, BN20: brain-cancer-specific module, *SCID* Structured Clinical Interview for DSM, *DT* Distress Thermometer

#### After the doctor–patient consultation, t2

After the doctor–patient consultation, the patients complete the above mentioned questionnaire for support needs a second time.

In addition, the Structured Clinical Interview (SCID) for DSM (Diagnostic and Statistical Manual of Mental Disorders) is conducted with the patients. The SCID is used to identify mental health conditions [20]. The interviewer asks certain questions and codes the answers in order to be able to make a diagnosis at the end. The SCID is widespread, reliable, and valid [20]. The study nurses have been trained beforehand to conduct the interviews. Each controversial case will be discussed in a telephone conference with a supervisor (Table 1).

#### Follow-up, t3

Three months later, the patients will complete the EORTC QLQ-C30 + BN20 questionnaire and again an adapted version of the self-developed questionnaire (Table 1) in order to assess the proportion of patients receiving adequate care.

#### Training of study personnel and monitoring

During the start-up phase of the study, the research assistants and study nurses have been trained to conduct the SCID interview reliably. They also learned about the assessment procedures at t1–t3. Furthermore, worksheets and case report forms (CRFs) have been developed, evaluated, and adapted after a pilot test in September 2019. All centers will be initiated and monitored by the project manager of the study. Frequently (monthly) conducted telephone conferences regarding the SCID interview and study meetings planned twice a year will help to solve any problems.

### Statistics

#### General considerations for data analysis and statistical methods of the study

Through cluster randomization, the study can be better implemented into clinical routine.

Broadly selected inclusion criteria ensure that as many patients as possible are reached. The aim is to include not only highly selected patients.

#### Data management

Data will be derived from patients’ responses to questionnaires, worksheets completed by study nurses and research assistants, as well as from medical records. All data will be collected specifically for research purposes.

All person-identifying data are avoided in the data set and code numbers used instead. A study code will be assigned to each subject in the respective study center. A copy of all worksheets and completed questionnaires using the code numbers will be sent to the statistical center (IMBEI) in Mainz. Only pseudonymized data will be handed over. All study data will be stored for 10 years after completion of the study in the respective center, and only the study team will have access to the data. In Mainz the central data processing and evaluation will be done. Pseudonymized data will be stored in a password-protected electronic database, which is stored in a locked server only accessible for staff members of the IMBEI with access authorization.

#### Sample size

Based on previous studies [21] we expect that in the control group only about 15% of mentally stressed patients receive psychosocial care compared to an expected 25% in the intervention group.

Due to the fact that this is a cluster-randomized study, more patients have to be included in the study.

The sample size calculation is based on recommendations by Hayes and Bennett, who developed several scenarios for cluster-randomized studies and provided formulas for the calculation of the cluster effect. The intracluster correlation coefficient (ICC) in a comparable, cluster-randomized study with 13 clusters was between 0.03 and 0.19 depending on the outcome [21]. However, Donner and Klar (2004) warn of overestimating the stability and size of an ICC. Therefore, we assume an ICC of 0.005. We further assume that 50 patients will be included in the study in each clinic and that the clusters will be of the same size. In this scenario (ICC 0.005), 12 clinics with a total of *n* = 616 patients are required to demonstrate the expected intervention effect (25% vs 15%) with α of 0.05 and a power of 80%.

#### Control of bias and confounding

Potential confounders (hospital organization, influence of individual doctors, local conditions affecting care, and resources) are controlled by randomization. However, since cluster randomization may result in imbalances of baseline differences, these variables are also documented and analyzed.

Through the use of standardized questionnaires, the possibility of information bias is reduced.

It should be considered that support needs could be awakened by participation in this study. For this reason, questions are asked before and after the doctor’s conversation in order to be able to identify possible changes. However, since screening for psychosocial stress is required in all cancer patients according to the German guideline on psycho-oncological diagnostics and treatment, we do not consider it ethically justifiable to establish a third study arm that does not ask about the burden and the care desired.

#### Statistical analysis

The analysis of the primary outcome “proportion of adequate care for patients with increased psychosocial stress” will be based on hierarchical models for cluster-randomized studies (with random intercept for the clinics).

Due to the limited number of clinics, the following variables will also be adjusted: sex, age, ECOG stage, initial or recurrent disease, neurological deficit, time since initial diagnosis, living alone vs in partnership. The analyses are performed according to intent to treat. The aim is to reduce a possible bias by selective drop-out. The secondary endpoints are also calculated using mixed models.

### Ethics approval

The study is performed in accordance with national law, institutional ethics standards, and the Declaration of Helsinki after approval of the study protocol by the local ethic committees (number of the first approval 837.179.17 (11013)). All centers participating in the study obtained ethics approval before recruiting patients. All patients provide written informed consent prior to data assessment.

The study has been registered in the German Clinical Trials Register (DRKS00018079).

## Discussion

The authors hypothesize that the currently outsourced psychosocial conversation will be integrated back into the doctor–patient conversation. Doctors will have to integrate the structured questions according to a standard operation procedure (SOP) into the conversation and additionally ask more specific questions. There may be fears that the conversation will be extended because sensitive issues will also be mentioned. However, the authors assume that, first, this will not be the case for all patients and, second, after a more intensive discussion at the beginning, the subsequent discussions will be less time-consuming. The burden on outpatient nursing staff will be reduced by eliminating the need to frequently collect questionnaires and explain why they have to be completed again, so that a high level of acceptance is expected. Relatives might also be relieved because they frequently assist the patients when completing self-assessment questionnaires.

### Risk factors and control procedures

The following points could be identified as possible risk factors:

- The questionnaire and the questions posed by the doctors address sensitive issues, which can be stressful for both patients and doctors. In addition, there may be an increased need for care because the study could reveal possible undersupply [22].
- Cluster randomization is used in the study. Since the clinics are regarded as clusters, the “personnel effect” must be taken into account and have to be considered in the interpretation. On the other hand—and this is of considerable importance for achieving the planned recruitment targets—cluster randomization is intended to reduce a possible hesitant recruitment of patients. Cluster randomization reduces the effort, since there is no need to act, document, and evaluate according to two different protocols (arm A, arm B) in a single center. In addition, patients with single randomization sometimes have concerns about participating in a study, which is reduced by this type of randomization [23]. Recruitment problems often arise because patients are sometimes more hesitant to participate in randomized trials. In this study, patients do not need to be told that they will be randomized if they participate in the study, as there is only one active study arm within a clinic. Furthermore, the planning of an early intermediate meeting 3 months after the start of the study serves to identify and solve problems.
- Since patients from all disease stages of a HGG (initial diagnosis, follow-up, and recurrent disease) are included, a survival bias may be the consequence. However, the authors point out that the questions were developed for this patient population (including all three stages) and that there were no differences in the content of relevant topics in the subpopulations leading to results transferable to clinical reality.
- The study protocol, standardized instruments, and the use of SOPs, as well as the consistent use of study personnel and targeted training of study personnel, reduce information and interviewer bias; on the other hand, the personnel effect is strengthened by this.
- Informed consent is of course required, probably leading to a selection bias: patients who decline to be in the study are not interviewed/recorded and are therefore not evaluated. Due to the few inclusion criteria and the “single-arm structure” of the study per center, the barriers to participate may be limited. This allows patients to participate in the study who often do not meet the inclusion criteria, which minimizes the selection bias. Patients who still reject are anonymously documented in order to clearly represent the selection bias.

### Practical feasibility

Through pragmatic economic project planning the study already simulates clinical routine. For example, low-threshold inclusion criteria should include as many patients as possible, including those in a reduced general condition. Furthermore, the cluster randomization should lead to a standardized intervention strategy in the clinics (minimization of effort). In this way, after final evaluation, the results should be transferable to practice. The changed recording of support needs will reduce the burden on patients, relatives, and nursing staff. The authors also expect an improvement in the doctor’s consultation. Since the question “How are you?” is routinely asked during the patient–doctor conversation, it should be possible (if the study is positive) to intensify and expand the conversation by three or four further questions. Due to the shortness of the structured assessment, there is practically no bureaucratic effort for doctors and/or patients, so we expect a good acceptance. The aim of the study is to develop a transferable manual that clearly outlines the procedure and implementation, structuring of the doctor–patient conversation as well as the interpretation of the results for the treating colleagues. Then the items or screening questions that result from the study for an interview for the doctor–patient conversation can be directly implemented by neuro-oncologists, general practitioners, and radio-oncologists as well as all other participating disciplines.

In the longer term, the result obtained in this study could therefore be applied to patients with other tumor entities, e.g., patients with brain metastases.

#### Study status

The study is ongoing. The project started on 01 June 2019 and patient enrollment started in September 2019 and is expected to end in November 2021. German Clinical Trials Register, DRKS00018079. Protocol **v**ersion 4.0 (01/11/2018).

## Data Availability

Data and materials are available upon direct request.

## Abbreviations

CAU: Care as usual
CRF: Case report form
DSM: Diagnostic and Statistical Manual for Mental Disorders
DT: Distress Thermometer
ECOG: Eastern Co-operative of Oncology Group
EORTC: European Organization of Research and Treatment of Cancer
EORTC-QLQ C30: European Organization of Research and Treatment of Cancer Quality of Life Core Questionnaire
EORTC-QLQ BN20: European Organization of Research and Treatment of Cancer brain cancer-specific module
H: High-grade glioma
ICC: Intracluster correlation coefficient
KPS: Karnofsky Performance Status
NANO: Neurologic Assessment in Neuro-Oncology
RANO: Response Assessment in Neuro-Oncology
SCID: Structured clinical interview for DSM
SOP: Standard operation procedure
